# Survey data on work environments and productivity of academic staff of selected public universities in Nigeria

**DOI:** 10.1016/j.dib.2018.06.074

**Published:** 2018-06-28

**Authors:** Odunayo Salau, Rowland Worlu, Adewale Osibanjo, Anthonia Adeniji, Olumuyiwa Oludayo, Hezekiah Falola

**Affiliations:** Covenant University, Nigeria

**Keywords:** Meaningful work, Supportive management, Physical Milieu, Growth opportunity, Trust

## Abstract

The article presented a survey data on work environment predictors and productivity of selected academic staff of selected public universities in Southern, Nigeria. The study adopted a quantitative approach with a survey research design to establish the major determinants of work environments in the selected public universities. Data was analysed with the use of structural equation modelling and the field data set is made widely accessible to enable critical or a more comprehensive investigation. The findings identified meaningful work and growth opportunities as predictive factors for maximizing productivity in the sampled institutions.

**Specification table**

**Table:** 

**Subject area**	Business, Management
**More Specific Subject Area:**	Strategic human resource management
**Type of Data**	Primary data
**How Data was Acquired**	Through questionnaire
**Data format**	Raw, analyzed, Inferential statistical data
**Experimental Factors**	Population comprises academic staff of selected public universities in Southern, Nigeria.
**Experimental features**	The researcher-made questionnaire which contained data on work environments and productivity.
**Data Source Location**	Lagos, Nigeria
**Data Accessibility**	Data is included in this article

**Value of data**

● The data can be used by government and other stakeholders to make decisions that in the long-run would lead to maximum productivity in our tertiary institutions.
● The data can be used to advise government on the importance of healthy work environments and how it can be beneficial to the overall productivity of the institutions.
● The data provides information on how different work environment attributes can interact effectively to enhance productivity and sustaining greater commitment.

## 1 Data

Creating healthy work environment has become an important success factor in any competitive and demanding environments such as the educational sector. The study is quantitative in nature and data were retrieved from staff and management of the sampled institutions. The use of semi structured questionnaire was adopted to elicit information from selected respondents. The use of questionnaire was relevant because the sample was large enough to accommodate statistical analysis and integrate the socio-demographic and work environment variables. An extensive list of items in the questionnaire was developed to understand the nature and the type of work environments provided by the sampled institutions. Work Environment was measured using items adapted from previous studies [2], [3], [5], [6], [7] while productivity was measured through the scale items by [9].

**Fig. 1:**
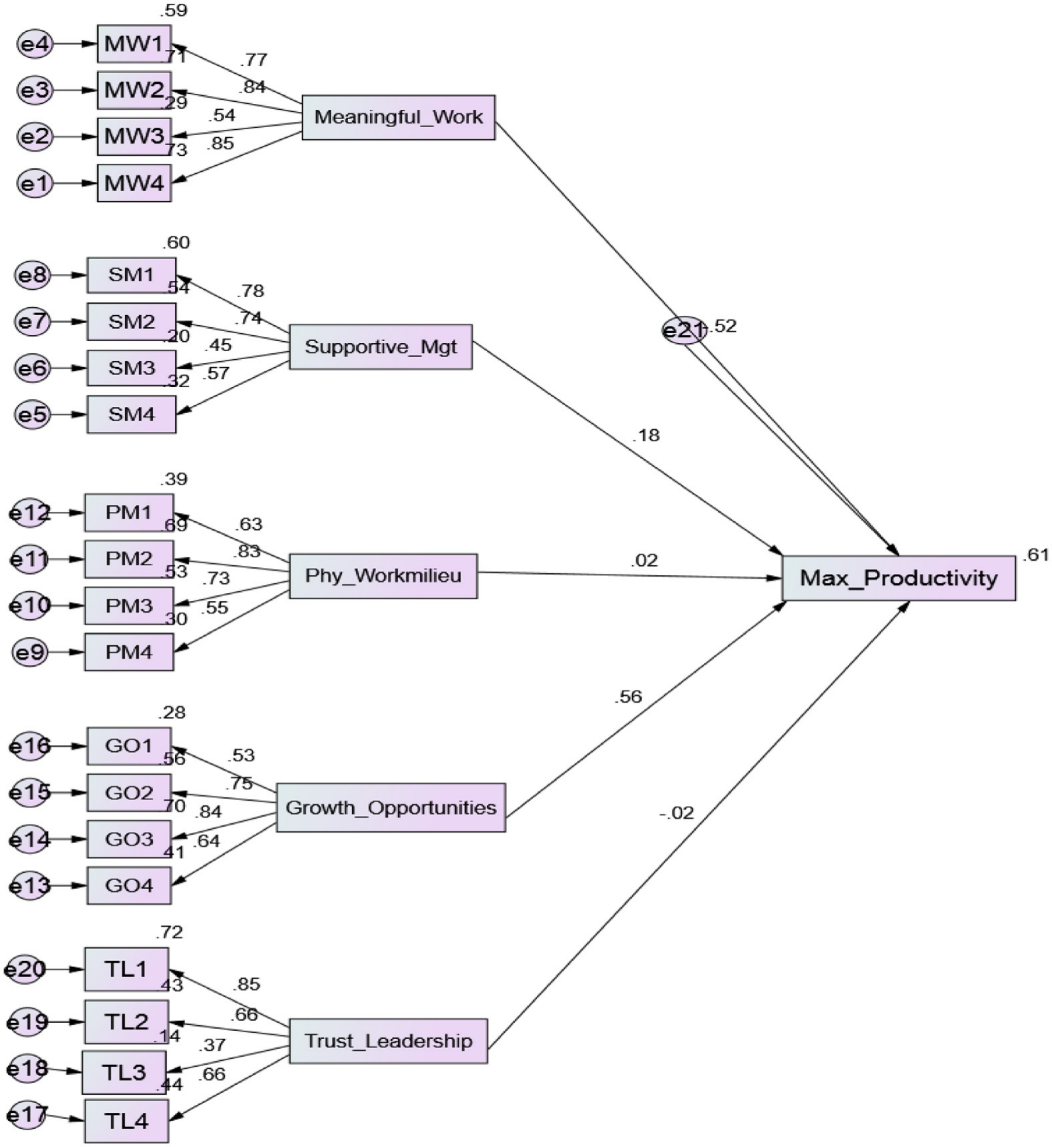
Regression weights of the variables.

The variables considered include: the extent of meaningful work, degree of physical work-milieu, trust leadership, growth opportunities and the levels of supportive management [[1], [8], [10]]. The returned copies of questionnaire were edited to check for and minimize errors. When done, copies of questionnaire with incomplete information were discarded while completed ones were coded for analysis. The quantitative data was analysed and the results of measurement (CFA) and model (SEM version 22.) tests were reported in Table 1 and a detailed description of healthy work environment (WE) was provided in Fig. 1.

**Table 1:** Demonstrated Confirmatory Factor Analysis (CFA) of the constructs.

**Indicators**	**Loading**	**Indicator reliability**	**Error variance**	**Sum of variance**	**Compose reliability**	**Ave. variance estimated**
	**>0.7**		**<0.5**		**>0.8**	**>0.5**
	**Meaningful Work (MW)**					
MW1	0.7660	0.5868	0.4132	1.6843	0.8424	0.5789
MW2	0.8440	0.7123	0.2877			
MW3	0.5360	0.2873	0.7127			
MW4	0.8540	0.7293	0.2707			
	**Physical Work-Milieu (PM)**					
PM1	0.6260	0.3919	0.6081	2.0779	0.8232	0.5805
PM2	0.8320	0.6922	0.3078			
PM3	0.7310	0.5344	0.4656			
PM4	0.5510	0.3036	0.6964			
	**Trust Leadership (TL)**					
TL1	0.8490	0.7208	0.2792	2.2681	0.8403	0.5330
TL2	0.6590	0.4343	0.5657			
TL3	0.3740	0.1399	0.8601			
TL4	0.6610	0.4369	0.5631			
	**Growth Opportunities (GO)**					
GO1	0.5320	0.2830	0.7170	2.0446	0.8883	0.5889
GO2	0.7470	0.5580	0.4420			
GO3	0.8380	0.7022	0.2978			
GO4	0.6420	0.4122	0.5878			
	**Supportive Management (SM)**					
SM1	0.7760	0.6022	0.3978	2.3294	0.8333	0.5176
SM2	0.7380	0.5446	0.4554			
SM3	0.4510	0.2034	0.7966			
SM4	0.5660	0.3204	0.6796

To support the measurement model, the use of structural equation modelling (SEM) was adopted to explain the relationship between sets of observed and latent variables [4]. The conditions for SEM indicate firstly, that all scale items such as NFI, CFI, GFI and IFI are significant when it exceeds the minimum value criterion of 0.90.

**Table:** 

Indicators	GFI	CFI	NFI	IFI	CMIN/DF	RMSEA
Benchmark	≥ 0.90	≥ 0.90	≥ 0.90	≥ 0.90	≤ 3.00	≤ 0.08
Result	.952	.937	.963	.953	2.522	0.059

Note: The results of measurement and structural model indicate that conditions of factor loadings and SEM indices were met.

**Fig. 2:**
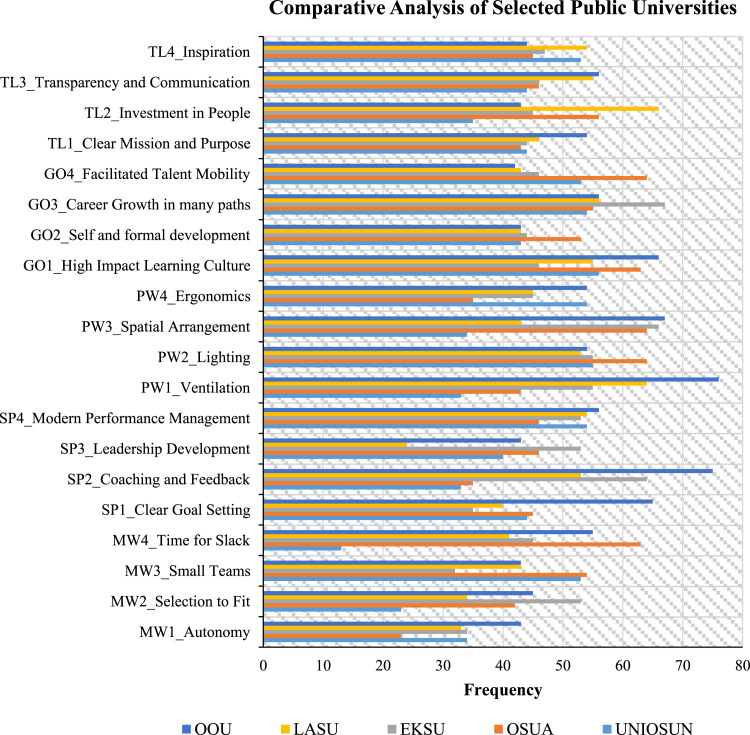
Comparative analysis of selected public universities.

A comparative analysis of the selected public (state) universities was also capture as presented in Fig. 2.

## 2 Experimental design, materials and methods

Of the 250 copies of questionnaire distributed, 224 responses were received, resulting in a response rate of 89.6%. Academic staff of selected five public universities (Olabisi Onabanjo University, Ekiti State University, Lagos State University, Osun State University, and Ondo State University of Science and Technology), South-west, were represented in this study. The use of questionnaire was used to collect quantitative data on the assessment of work environments and productivity among University academic staff. Participants were requested to respond to items in a self-administered, quick-answer, structured (close-ended) and unstructured (open-ended) copies of questionnaire. Primary data were collected using questionnaire.

The choice of using questionnaire for collecting data from the cross section of the sampled population depend on the variables that were measured, the source and the resources available. The collection of data for this study was achieved by requesting and obtaining relevant data provided directly by the staff of the sampled firms to maximise timeliness and data accuracy. The researchers established and maintained good relationships with the sampled respondents in order to obtain a good response rate. In order to maximise return rates, the items in the questionnaire were designed to be as simple and clear as possible, with targeted sections and questions. The questionnaire contained structured questions (with multiple choice and open-ended questions) which were used to encourage respondents to reply at length and choose their own focus to some extent.

Data collection for this study involved a combination of different activities. The first step was to recruit and train Field Assistant to administer the questionnaire alongside the researchers. Five (5) people were recruited and trained on questionnaire administration and other social issues associated with it. They (the research assistant) were also trained to understand the study questionnaire, the processes necessary for successful administration, their allotment in the administration, how to solve probable challenges they may encounter. The training was to ensure that all assistant had a thorough understanding of the concept, the place, the people and the instrument before proceeding into the field.

The responses from multiple choice and open-ended questions were thematized to facilitate clearly identifiable database entry and analysis. The data collected were extracted and converted into electronic format and subsequently coded by assigning numerical values to the responses. The study indicated a meaningful relationship between work environments and productivity among academic staff of the selected institutions. The collected data were coded and analysed using SPSS version 22. Data was analysed applying descriptive (bar chart) and inferential statistical tests such as structural equation modelling (SEM). Importantly, the study participants were academic staff of the sampled public universities; participants have worked with the institution(s) for a minimum period of 3 years and finally, participants were accessible as at the time of the survey and interviews. The researchers ensured that respondents were well informed about the background and the purpose of this research and they were kept abreast with the participation process. Respondents were offered the opportunity to stay anonymous and their responses were treated confidentially.
