# Supplementary material for: Survey data on work environments and productivity of academic staff of selected public universities in Nigeria
Source: Data Brief. 2018 Jun 28;19:1912–7. doi: 10.1016/j.dib.2018.06.074 (PMC6141789; doi:10.1016/j.dib.2018.06.074)
Supplement: Supplementary file 1 — Supplementary material [file mmc1.docx]

**DECLARATION OF INTEREST FORM**

**COMPARATIVE ASSESSMENT OF DATA OBTAINED FOR MAXIMIZING HEALTHY WORK ENVIRONMENTS AND PRODUCTIVITY AMONG ACADEMIC STAFF OF SELECTED PUBLIC UNIVERSITIES**

Odunayo **SALAU**; Covenant University

[odunayo.salau@covenantuniversity.edu.ng](mailto:odunayo.salau@covenantuniversity.edu.ng)

Rowland WORLU; Covenant University

rowlandworlu@covenantuniversity.edu.ng

Adewale OSIBANJO; Covenant University

ade.osibanjo@covenantuniversity.edu.ng

Anthonia ADENIJI; Covenant University

[anthonia.adeniji@covenantuniversity.edu.ng](mailto:anthonia.adeniji@covenantuniversity.edu.ng)

Olumuyiwa OLUDAYO Covenant University

[olumuyiwa.oludayo@covenantuniversity.edu.ng](mailto:olumuyiwa.oludayo@covenantuniversity.edu.ng)

Hezekiah FALOLA Covenant University

hezekiah.falola@covenantuniversity.edu.ng

We, the Authors of paper entitled above certify that we have seen and approved the final version of the manuscript being submitted. This is an original work and has not received prior publication and is not under consideration for publication elsewhere. It is also important to state that there is no financial/personal interest or belief that could affect our objectivity and to prevent ambiguity, we humbly want to state explicitly that there is no conflicts of interest as regards the review and publication of this paper.

Thank you.

SALAU Odunayo Paul

*Signed*
